# Long-term kidney function in children with Wilms tumour and constitutional *WT1* pathogenic variant

**DOI:** 10.1007/s00467-021-05125-5

**Published:** 2021-10-04

**Authors:** Maria Pia Falcone, Kathryn Pritchard-Jones, Jesper Brok, William Mifsud, Richard D. Williams, Kayo Nakata, Suzanne Tugnait, Reem Al-Saadi, Lucy Side, John Anderson, Catriona Duncan, Stephen D. Marks, Detlef Bockenhauer, Tanzina Chowdhury

**Affiliations:** 1 Department of Paediatric Oncology Great Ormond Street Hospital, UCL Institute of Child Health, NIHR Great Ormond Street Hospital Biomedical Research Centre, London, UK; 2 Paediatric Residency Program, University of Foggia, Foggia, Italy; 3 Dept. of Paediatric Haematology and Oncology, Rigshospitalet, Copenhagen University Hospital, Copenhagen, Denmark; 4 Dept. of Histopathology, Great Ormond Street Hospital for Children NHS Foundation Trust, London, UK; 5 Dept. of Clinical Genetics, Great Ormond Street Hospital for Children NHS Foundation Trust, London, UK; 6 Dept. of Paediatric Nephrology, Great Ormond Street Hospital for Children NHS Foundation Trust, London, UK; 7 UCL Department of Renal Medicine, London, UK

**Keywords:** Wilms tumour, *WT1* pathogenic variant, Kidney function

## Abstract

**Background:**

Wilms tumour (WT) survivors, especially patients with associated syndromes or genitourinary anomalies due to constitutional *WT1* pathogenic variant, have increased risk of kidney failure. We describe the long-term kidney function in children with WT and *WT1* pathogenic variant to inform the surgical strategy and oncological management of such complex children.

**Methods:**

Retrospective analysis of patients with WT and constitutional *WT1* pathogenic variant treated at a single centre between 1993 and 2016, reviewing genotype, phenotype, tumour histology, laterality, treatment, patient survival, and kidney outcome.

**Results:**

We identified 25 patients (60% male, median age at diagnosis 14 months, range 4–74 months) with *WT1* deletion (4), missense (2), nonsense (8), frameshift (7), or splice site (4) pathogenic variant. Thirteen (52%) had bilateral disease, 3 (12%) had WT-aniridia, 1 had incomplete Denys-Drash syndrome, 11 (44%) had genitourinary malformation, and 10 (40%) had no phenotypic anomalies.

Patient survival was 100% and 3 patients were in remission after relapse at median follow-up of 9 years. Seven patients (28%) commenced chronic dialysis of which 3 were after bilateral nephrectomies. The overall kidney survival for this cohort as mean time to start of dialysis was 13.38 years (95% CI: 10.3–16.4), where 7 patients experienced kidney failure at a median of 5.6 years. All of these 7 patients were subsequently transplanted. In addition, 2 patients have stage III and stage IV chronic kidney disease and 12 patients have albuminuria and/or treatment with ACE inhibitors. Four patients (3 frameshift; 1 *WT1* deletion) had normal blood pressure and kidney function without proteinuria at follow-up from 1.5 to 12 years.

**Conclusions:**

Despite the known high risk of kidney disease in patients with WT and constitutional *WT1* pathogenic variant, nearly two-thirds of patients had sustained native kidney function, suggesting that nephron-sparing surgery (NSS) should be attempted when possible without compromising oncological risk. Larger international studies are needed for accurate assessment of *WT1*genotype-kidney function phenotype correlation.

## Introduction

Wilms tumour (WT) or nephroblastoma is an embryonal tumour and the most common kidney tumour in childhood. It affects one in 10,000 children and accounts for about 5% of all childhood cancers [1–3]. Survival of WT has improved significantly and the cure rate is approximately 90% when adhering to optimal treatment [4]. However, the treatment and the genetic conditions occasionally associated with the disease result in additional health challenges among survivors. Kidney failure in particular is a concerning outcome. In the National Wilms Tumor Study (NWTS), the 20-year cumulative overall incidence of kidney failure was 1.3% for unilateral WT patients and 15% for bilateral WT [5].

Significantly higher rates of kidney failure were found among patients with WAGR syndrome (WAGR), Denys-Drash syndrome (DDS), and those with associated male genitourinary (GU) anomalies, all related to constitutional *WT1* pathogenic variants [6, 7]. The rate of deterioration of kidney function varies according to the syndrome, with a much earlier onset of kidney failure described in children with DDS (due to intragenic *WT1* pathogenic variants) than in those with a complete deletion of one allele of *WT1*, as in WAGR syndrome [8]. Frasier syndrome, due to *WT1* splicing pathogenic variant, has an intermediate rate of decline in kidney function and a much lower risk of WT [9, 10].

It has been reported that 74% of Denys-Drash patients, 36% of WAGR patients, and 7% of hypospadias or cryptorchidism patients had kidney failure at 20 years of follow-up, compared with only about 1% of non-syndromic children [5].

The phenotypic spectrum associated with constitutional *WT1* pathogenic variant is broad. We await prospective studies to determine more accurately the proportion of patients with *WT1* pathogenic variant who have unilateral WT without associated GU abnormalities. Two previous studies that sequenced the *WT1* gene in non-syndromic children with WT found a very low percentage in the absence of at least bilateral disease [6, 11]. Therefore, relying on phenotype alone to identify individuals with WT who have constitutional *WT1* pathogenic variant may be challenging. Factors that may indicate that an individual with normal phenotype is carrying a constitutional *WT1* pathogenic variant are as follows: bilateral disease, diagnosis with WT before the age of 1 year, stromal-predominant histology, and intralobar nephrogenic rests (ILNR) [12–14]. Among such patients, with incomplete clinical features of *WT1*-related syndromes and in whom *WT1* missense or stop pathogenic variants are found, the impact of pathogenic variant type on the expected rate of deterioration of kidney function is currently unclear.

A well-known oncological dilemma is the balanced decision between either complete resection of WT to optimize tumour control, or the performing of nephron-sparing surgery (NSS) for syndromic patients to preserve kidney function. When feasible, it is now standard practice that NSS should be attempted at the time of WT resection in children with bilateral tumours, syndromic features, and those with other predisposing factors. Due to the higher risk of relapse in the contralateral kidney, the maximum possible parenchymal reserve capacity should be preserved, in order to prevent or postpone kidney failure [14]. Prior knowledge of the presence of a constitutional *WT1* pathogenic variant and its subtype may have important implications in predicting the risk and rate of deteriorating function of the remaining nephrons [15, 16].

The aim of our study is to describe the long-term kidney function of children with WT and constitutional *WT1* pathogenic variant in relation to their phenotype, genotype, and treatment received. These findings could guide clinical management in the future of children with similar clinical and genetic features, through a greater understanding of the longevity of their clinically useful kidney function.

## Methods

### Patients

We identified retrospectively from hospital records and the oncology departmental database of patients with kidney tumours, cases of patients with co-existing WT and constitutional *WT1* pathogenic variant who received treatment between 1993 and 2016 at Great Ormond Street Hospital for Children NHS Foundation Trust (GOSH), UK. *WT1* pathogenic variant testing was done at different points for each patient as directed by the clinical presentation. This could be at diagnosis, during treatment, or at follow-up particularly in the older cases. Patients with WAGR and DDS were included. WAGR patients were defined as those with the full complement of WT, aniridia (AN), GU malformations, and intellectual disability (MIM#194072). DDS patients were defined as having WT, nephropathy presenting as persistent proteinuria or overt nephrotic syndrome, and GU anomalies (MIM#194080) [17, 18]. WT patients with only some of these phenotype anomalies were categorized by their specific features (AN, diffuse mesangial sclerosis (DMS), and GU malformations).

We collected data on patient and tumour demographics which comprised patient gender, age at diagnosis, and phenotypic anomalies; tumour histology, laterality, multifocality, tumour volume at diagnosis and after pre-operative treatment; and treatment details of chemotherapy, type of surgery, and radiation received.

All patients had pre-operative chemotherapy and tumour histological subtype was according to the revised SIOP classification [19]. For tumours classified initially according to other protocols, a second pathological review was performed by an expert pediatric pathologist (WM) for the purposes of this study. Tumours were also categorized according to the presence or absence of rhabdomyoblastic differentiation and intralobar and perilobar nephrogenic rests (ILNR and PLNR, respectively).

### Pathogenic variant detection

All *WT1* pathogenic variants and deletions were identified from peripheral blood lymphocytes by an NHS genetic diagnostic service, using Sanger sequencing, MLPA (multiplex ligation-dependent probe amplification), and, in older cases, FISH (fluorescent in situ hybridization). Non-syndromic patients with WT in our service were selected for constitutional *WT1* pathogenic variant testing at the discretion of the treating clinician based on previously published criteria suggesting a higher risk of such pathogenic variant [13] or when they had some features of DDS in the absence of urogenital malformation (persistent hypertension or proteinuria).

Patients were classified according to the type of *WT1* pathogenic variant: deletion, missense, nonsense, frameshift, and splice site.

### Kidney function

We assessed the following data at diagnosis and at the last follow-up: kidney function with serum creatinine and estimated glomerular filtration rate (eGFR), urine albumin to creatinine ratio, blood pressure, and use of anti-hypertensiveand/oranti-proteinuric medications.

The GFR was estimated using the revised Schwartz formula: eGFR (ml/min/1.73 m^2^) = k × height (cm)/serum creatinine (μmol/l) with a k value of 33 [20, 21]. If the height of the patient was missing, it was estimated from the percentile growth chart of the patient. The eGFR was used to stage the kidney function of the patients according to standard criteria [22]. Albuminuria was defined as a urine albumin to creatinine ratio above the age-adjusted normal range.

WT patients were categorized as having kidney failure if they received chronic kidney replacement therapy with dialysis or transplantation. The malignancy was regarded as the cause of kidney failure if the patient needed surgical removal of all the kidney tissue as a result of widespread/progressive bilateral WT or a relapse in the solitary kidney that required nephrectomy.

### Statistical analysis

Numerical variables were summarized as medians and standard deviation as the data followed a non-parametric distribution. Categorical and ordinal variables were described as relative frequencies. Medians and frequencies were compared using Mann-Whitney’s U test and the Kruskal-Wallis test respectively. All tests were two-sided and a p value = 0.05 was considered significant. All statistical analysis was performed using SPSS© (version 24.0; SPSS Inc. Chicago, IL).

## Results

### Patient characteristics

We identified 26 patients with WT and constitutional *WT1* pathogenic variant and one patient with a complete phenotype of WAGR syndrome who had no documented analysis of *WT1* pathogenic variant but is assumed to have a constitutional deletion (Table 1). We excluded two patients, one with less than 6 months of follow-up from the date of diagnosis and one without available data on kidney function. Fifteen were male and median age at diagnosis of the whole cohort was 14 months (range 4–74 months).

**Table 1:** Germline *WT1* alterations in patients with Wilms tumour

Sex	Age at WT diagnosis (years)	Tumour laterality	Phenotype anomalies	*WT1* (P19544-1)^†^	*WT1* (P19544-7)^‡^	Phenotype anomalies	Exon/intron	References
*WT1* gene deletions								
F	1.2	U	AN	11p14.1-p11.2 del	11p14.1-p11.2 del	Aniridia	-	-
M	1.7	U	GU	11p13 del*	11p13 del*	Cryptorchidism limb deformity	-	-
M	2.2	B	WAGR	11p13 del	11p13 del	WAGR	-	-
M	4.0	U	**WAGR	-		WAGR	-	-
*WT1* gene missense								
M	1.1	U	GU	c.1214A>G, p.(His405Arg)	c.1433A>G, p.(His478Arg)	Cryptorchidism	Exon 9	Hu et al. [23]
F	1.5	U	DDS	c.1180C>T, p.(Arg394Trp)	c.1399C>T, p.(Arg467Trp)	Diffuse mesangial sclerosis-severe hypertension	Exon 9	Pelletier et al. [17]
*WT1* gene nonsense								
M	1.2	B	N	c.901C>T, p.(Arg 301*)	c.1120C>T, p.(Arg374*)	N	Exon 7	Schumacher et al. [24]
F	1.6	B	N	c.938C>A, p.(Ser313*)	c.1157C>A, p.(Ser386*)	N	Exon 7	Schumacher et al. [24]
F	0.9	B	N	c.1021C>T, p.(Gln341*)	c.1240C>T, p.(Gln414*)	N	Exon 7	Royer-Pokora et al. [25]
F	1.2	B	N	c.1021C>T, p.(Gln341*)	c.1240C>T, p.(Gln414*)	N	Exon 7	Royer-Pokora et al. [25]
F	1.9	B	N	c.1084C>T, p.(Arg 362*)	c.1303C>T, p.(Arg435*)	N	Exon 8	Clarkson et al. [26]
M	2.5	B	GU	c.1084C>T, p.(Arg362*)	c.1303C>T, p.(Arg435*)	Cryptorchidism/ hypospadias/ micropenis	Exon 8	Clarkson et al. [26]
M	0.5	B	GU	c.1168C>T, p.(Arg 390*)	c.1387C>T, p.(Arg463*)	Cryptorchidism	Exon 9	Shibata et al. [27]
F	0.8	U	N***	p.(Arg390*)	p.(Arg463*)	N***	Exon 9	Shibata et al. [27]
*WT1* gene frameshift								
M	0.3	U	GU	c.895delG, p.(Asp299fs)	c.1114delG, p.(Asp372fs)	Cryptorchidism	Exon 7	Royer-Pokora et al. [25]
F	0.7	B	N	c.256delG, p.(Glu86fs)	c.475delG, p.(Glu159fs)	N	Exon 1	-
M	0.7	U	GU	c.928-929delCT, p.(Leu310fs)	c.1147- 1148delCT, p.(Leu383fs)	Cryptorchidism/ hypospadias	Exon 7	-
M	0.9	B	GU	c.957delG, p.(Lys320fs)	c.1176delG, p.(Lys393fs)	Cryptorchidism	Exon 7	-
M	1.5	U	GU	c.1015delC, p.(His339fs)	c.1234delC, p.(His412fs)	Cryptorchidism	Exon 7	-
M	5.2	U	GU	c.157-166del10, p.(Gly53fs)	c.376-385del10, p.(Gly126fs)	Cryptorchidism/ hypospadias	Exon 1	-
M	0.6	B	GU	c.128del, p.(Pro43fs)	c.347del, p.(Pro116fs)	Cryptorchidism	Exon 1	-
*WT1* gene splice site								
F	0.6	B	N	c.895-1G>A	c.1114-1G>A	N	Intron 6	Lee et al. [28]
M	0.8	U	GU	c.668+2T>G	c.887+2T>G	Cryptorchidism/ hypospadias	Intron 3	-
F	1.4	B	N	c.1045+1G>A	c.1264+1G>A	N	Intron 7	-
M	6.2	U	N****	c.1228+5G>A	c.1447+5G>A	N****	Intron 9	Bruening et al. [29]

*Deletion did not extend to the *PAX6* gene

**Patient with a complete phenotype of WAGR syndrome who had no documented analysis of *WT1* but is assumed to have a constitutional deletion

***This patient presented with unilateral Wilms tumour but subsequently developed kidney dysfunction that indicated *WT1* pathogenic variant testing and was also subsequently found to have contralateral lesions

****This patient presented at an older age with Wilms tumour and steroid-resistant nephrotic syndrome without GU abnormalities

^†^Coding DNA and translated protein variants in this column and in the text are numbered relative to the first base of the ATG initiation codon at nucleotide 399 in NCBI reference sequence NM_024426.6, corresponding to UniProt canonical WT1 protein isoform P19544-1

^‡^Variants in this column are numbered relative to the first base of the upstream CTG initiation codon at nucleotide 180 in NCBI reference sequence NM_024426.6, corresponding to UniProt WT1 protein isoform P19544-7 (equivalent to NCBI NP_077744.4)

*U* unilateral, *B* bilateral, *AN* aniridia, *GU* genitourinary, *N* none, *DDS*Denys-Drash syndrome, *WAGR* Wilms tumour-aniridia

Fifteen (60%) patients (13 males, 2 females) had associated phenotypic anomalies: WAGR (n = 2; 8%); AN (n = 1; 4%); incomplete DDS (n = 1; 4%); GU malformations (n = 11; 44%) including cryptorchidism, hypospadias, and micropenis. In the remaining 10 (40%) patients, no phenotypic anomalies were found. In particular, no GU abnormalities were reported in any female patients (Table 1). One unusual male patient carried the splice site pathogenic variant associated with Frasier syndrome that prevents formation of the +KTS isoform of *WT1* but lacked any GU malformation. He presented at an older age with WT (74 months) and had albuminuria (albumin to creatinine ratio 140 mg/mmol) but normal creatinine and GFR. His kidney function deteriorated slowly over the subsequent 7 years at which time transplant was recommended. Another female patient had unilateral WT without other abnormal clinical features but presented with nephrotic syndrome 3 years after her WT nephrectomy. She developed stage 5 chronic kidney disease 5 years later, underwent second nephrectomy with evidence of nephroblastomatosis, and received a transplant 11 years later.

### *WT1* constitutional pathogenic variants

The patients were subdivided according to type of *WT1* pathogenic variant into the following subgroups (Table 2): large WAGR deletions encompassing the 11p13 region and entire *WT1* gene (4 patients; note one deletion did not extend into the *PAX6* gene); missense (2 patients); nonsense (8 patients); frameshift (7 patients); and splice site pathogenic variant (4 patients). The patient with full WAGR phenotype but no genetic testing is assumed to carry deletion of the 11p13 region.

**Table 2:** Clinical features of the patients with WT and *WT1* pathogenic variant

Characteristic	All patients	Type of *WT1* mutation				
		Deletion	Missense	Nonsense	Frameshift	Splice site
Patient (number)	25	4	2	8	7	4
Male	15 (60%)	3	1	3	6	2
Female	10 (40%)	1	1	5	1	2
Median age at diagnosis (months)/range	14	24 (14.0–47.5)	15 (12.6–17.6)	14 (5.5–29.6)	9 (3.8–62.9)	13 (7.5–74.3)
Median follow-up duration (years)/range	9	6 (1.0–9.9)	6 (4.2–7.5)	14 (1.2–21.9)	9 (1.5–12)	11 (6.7–19.4)
Phenotype, n (%)						
WAGR	2 (8%)	2	-	-	-	-
Aniridia	1 (4%)	1	-	-	-	-
DDS	1 (4%)	-	1	-	-	-
GU malformation	11 (44%)	1	1	2	6	1
No phenotype alteration	10 (40%)	-	-	6	1	3
Bilateral tumour, n (%)	13 (52%)	1	-	7	3	2
Tumour histology, n (%)*						
Total no. of tumours	36					
Mixed type	13 (36%)	2	2	5	3	1
Stromal type	17 (47%)	3	-	6	5	3
Regressive type	2 (6%)	-	-	-	1	1
Blastemal type	1 (3%)	-	-	-	-	1
Nephrogenic rest only	3 (8%)	-	-	2	1	-
Nephrogenic rest, n (%)	25 (70%)	5	2	9	4	5
ILNR, n (%)	19 (76%)	4	1	8	3	3
PLNR, n (%)	6 (17%)	-	2	1	1	2
Rhabdomyoblastic differentiation, n (%)	22 (61%)	1	-	9	8	4
Relapse, n (%)	3 (12%)	-	-	1	1	1
Haemodialysis, n (%)	7 (28%)	-	1	4	1	1
Kidney transplant, n (%)	7 (28%)	-	1	4	1	1
eGFR < 60 ml/min/1.73 m^2^ at last follow-up	2 (8%)	-	-	1	-	1
Albuminuria	9 (36%)	2	1	2	2	2
On anti-hypertensive drugs	10 (40%)	3	1	2	3	1

*Histological subtypes refer to 24 patients, 12 with bilateral disease (36 tumours)

The median age at WT diagnosis according to pathogenic variant type was 9 months (3.8–62.9) frameshift, 13 months (7.5–74.3) splice site, 14 months (5.5–29.6) nonsense, 15 months (12.6–17.6) missense, and 24 months (14.0–47.5) deletions for their respective pathogenic variant types.

We observed variation in the frequency of bilateral tumours by pathogenic variant type, 7/8 patients carrying nonsense pathogenic variants compared with frameshift (3/7), splice site (2/4), deletions (1/4), and missense (0/2), but this did not reach statistical significance (p = 0.192; Tables 1 and 2). Both missense variants affect functionally important residues in zinc finger 3, base-contacting residue Arg394, and zinc co-ordination residue His405.

### Tumour features

#### Tumour stage

Most tumours (n = 22/36; 61%) were abdominal stage 1. Three patients (12%) had metastases at diagnosis (two with lung metastases and one with liver metastases). Bilateral WT was observed in 13 (52%) patients, more frequently in females (7/10), than in males (6/15; p = 0.216).

#### Tumour histology

Tumour histology was available for review in 24 patients, including 12 patients with bilateral disease (total 36 tumours). According to the SIOP 2001 classification, 23 (92%) patients had tumours that were intermediate-risk histology, one patient had high-risk (blastemal-type), and one had WT where the histological classification was unknown. Of 36 tumours where histology was known, 32 (88%) were of intermediate-risk histology, 17 (47%) of which were stromal-type; 13 (36%) mixed type; and 2 (6%) regressive type, and one was of high-risk blastemal-type histology. No patient had low-risk histology or anaplastic tumour. Three tumours (8%) showed only ILNR.

Rhabdomyoblastic differentiation was seen in 22/36 (61%) of tumours. In the normal kidney parenchyma adjacent to the tumour, diffuse mesangial sclerosis (DMS) was observed in two patients and nephrogenic rests in 25/36 (69%) tumours, where 19 tumours had ILNR, six had PLNR, and none had both.

Tumour volume was available for 19 patients according to imaging (magnetic resonance imaging, computed tomography, or ultrasound). Of these, five had multifocal disease. After pre-operative chemotherapy, 11 (58%) had tumour volume reduction and one had stable disease. Seven (37%) patients had increased tumour volume all of whom showed rhabdomyoblastic differentiation and 5/7 were of stromal subtype.

### Treatment

All patients had pre-operative chemotherapy with 18 patients treated according to the SIOP 2001 protocol and seven according to similar previous national protocols. Among the 13 patients with bilateral tumours, two underwent up-front bilateral nephrectomies, three had bilateral NSS, and eight unilateral nephrectomy with contralateral NSS. Of the 12 patients with unilateral tumour, one had bilateral nephrectomies due to kidney failure, two underwent unilateral NSS, and nine had unilateral nephrectomy of whom two subsequently had metachronous relapse treated by contralateral nephrectomy in one case and contralateral NSS in the other. Ultimately, 21/25 (84%) patients retained some functioning kidney tissue.

Five (20%) patients received radiotherapy, with four patients receiving unilateral flank radiotherapy due to abdominal stage III WT, and one patient whole lung radiotherapy due to pulmonary metastases.

### Clinical outcome

**Fig. 1:**
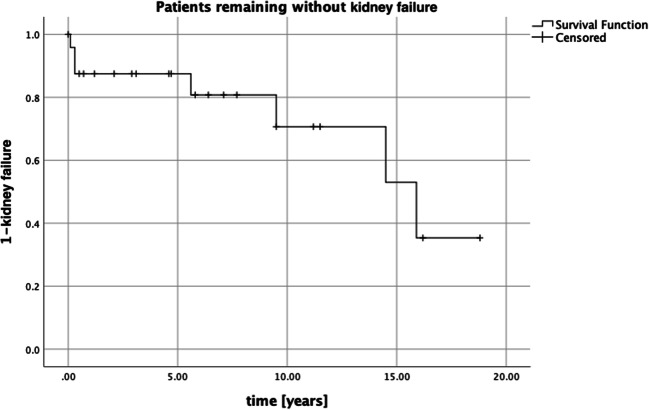
Kaplan-Meier graph of renal survival (time from diagnosis of WT to start dialysis). The overall renal survival for this cohort as mean time to start of dialysis was 13.38 years (95% CI: 10.3–16.4), where 7 patients reached kidney failure at a median of 5.6 years. All of the 7 patients were subsequently transplanted

Patient overall survival was 100%, with three patients in remission after disease relapse, and kidney survival was 72% at median follow-up of 9 years. Seven patients (28%) commenced chronic dialysis of which three were after bilateral nephrectomies (one patient had subsequent contralateral nephrectomy due to relapse). The median time between the diagnosis and the start of haemodialysis was 5.6 (0–16) years (Table 3). Kidney survival (time from diagnosis of WT to start dialysis) is shown by the Kaplan-Meier graph in Fig. 1. The overall kidney survival for this cohort as mean time to start of dialysis was 13.38 years (95% CI: 10.3–16.4), where 7 patients reached kidney failure at a median of 5.6 years. All of these seven patients were subsequently transplanted. Twelve patients had albuminuria and/or were prescribed ACE inhibitors. Two of these patients had albuminuria only, while among ten patients who were commenced on medication, six were being treated for hypertension and four were receiving anti-proteinuric medication. Two patients had stage III and stage IV chronic kidney disease (CKD). Four patients (3 frameshift; 1 *WT1* deletion) had normal blood pressure and kidney function without proteinuria at follow-up from 1.5 to 12 years (Table 4).

**Table 3:** Patients with WT and *WT1* pathogenic variant who required long-term dialysis

Sex	Laterality of tumour	Phenotype anomalies	*WT1* pathogenic variant	*WT1* (P19544-7)	Histologic subtype	Surgery	Relapse	Kidney transplant	Diagnosis–dialysis (years)	Follow-up (years)
M	B	GU	Nonsense	c.1387C>T, p.(Arg463*)	Stromal/mixed	UN+NSS	N	Yes	15.9	17.5
F	B	N	Frameshift	c.475delG, p.(Glu159fs)	Stromal/mixed	BN	N	Yes	0.3	11.6
F	U	N	Nonsense	p.(Arg463*)	Stromal	UN	N	Yes	9.5	21.9
M	U	GU	Splice site	c.887+2T>G	Stromal	UN*	Yes	Yes	5.6	10.4
F	U	DDS	Missense	c.1399C>T, p.(Arg467Trp)	Mixed/DMS	BN**	N	Yes	0.1	4.2
F	B	N	Nonsense	c.1303C>T, p.(Arg435*)	-	UN+NSS	N	Yes	14.5	14.9
F	B	N	Nonsense	c.1240C>T, p.(Gln414*)	Stromal	BN	N	Yes	0.3	3.0

*This patient had unilateral nephrectomy as first-line treatment and subsequent contralateral nephrectomy due to relapse

**Although the patient had a unilateral tumour, he received up-front bilateral nephrectomies due to kidney failure at diagnosis

*U* unilateral, *GU* genitourinary, *UN* unilateral nephrectomy, *NSS*nephron-sparing surgery

**Table 4:** Patients with WT and *WT1* pathogenic variant with normal kidney parameters after treatment

Sex	Laterality of tumour	Congenital abnormalities	*WT1* pathogenic variant	*WT1* (P19544-7)	Histologic subtype	Surgery	Relapse	Median follow-up (years)
M	U	GU	Frameshift	c.1114delG, p.(Asp372fs)	Stromal	UN	N	1.5
M	U	GU	Frameshift	c.1147- 1148delCT, p.(Leu383fs)	Mixed	UN	Y	12
F	U	AN	Deletions	11p14.1-p11.2 del	Mixed	NSS	N	3.3
M	U	GU	Frameshift	c.376-385del10, p.(Gly126fs)	Regressive	UN	N	11

*U* unilateral, *GU* genitourinary, *UN* unilateral nephrectomy, *NSS*nephron-sparing surgery

In each individual patient, we assessed the frequency of the following clinical/histologic factors suggestive of potential *WT1* pathogenic variant: age at diagnosis < 12 months, bilateral WT, GU malformation, ILNR, DMS, rhabdomyoblastic differentiation, albuminuria at diagnosis, and persistent hypertension after nephrectomy (Table 5). Nineteen of 25 patients had at least three or more of these clinical/histologic features, two patients had two characteristic features (GU malformation and ILNR), and the remaining four patients had only one feature, although two of these also had a *WT1*-associated syndrome.

**Table 5:** Prevalence of clinical and histologic factors associated with constitutional *WT1* pathogenic variant

	Features associated with constitutional *WT1* pathogenic variant								
	Age at diagnosis < 12 m	*WT1*-associated phenotype (WAGR, AN, GU, nephropathy)	Bilateral tumour	ILNR	Rhabdomyoblastic differentiation	Diffuse mesangial sclerosis	Albuminuria at diagnosis	Post-nephrectomy persistent hypertension	No. of features per patient
	X	X	X	X	X		X	X	7
			X	X	X	X	X	X	6
	X	X	X	X	X				5
	X		X		X		X	X	5
		X	X		X		X	X	5
	X	X		X	X				4
	X		X	X	X				4
	X		X	X	X				4
	X	X	X		X				4
	X	X			X				3
	X			X	X				3
	X	X			X				3
		X		X	X				3
			X	X	X				3
						X	X	X	3
		X		X	X				3
			X	X				X	3
		X	X	X					3
			X	X	X				3
		X		X					2
		X		X					2
			X						1
		X							1
		X							1
							X		1
Total with each feature	10	14	13	15	16	2	6	6	

## Discussion

This detailed study of 25 consecutively diagnosed patients with Wilms tumour and constitutional *WT1* pathogenic variant presenting to the largest childhood cancer centre in the UK over a 27-year period describes a clinical approach to recognizing such children and the potential for longevity of kidney function.

Our results confirm the previously described features observed in children with WT who carry a *WT1* pathogenic variant [6, 25] and emphasize how these can be variably present according to type of *WT1* pathogenic variant (Table 2) and by individual patient (Table 5). The small cohort size, due to the rarity of these patients, does not provide sufficient power for formal statistical analysis of *WT1*genotype-phenotype correlations.

Previous descriptions of WT in children with underlying constitutional *WT1* pathogenic variant have emphasized the association with GU malformation [29]. This is in line with the established role of *WT1* in normal GU development [30, 31]. Despite this, in our study, 10 (40%) patients had no GU abnormalities, and in particular, no GU malformations were reported in female patients. Among the four patients with large deletions encompassing the entire *WT1* gene, one did not have AN as their deletion did not encompass the *PAX6* gene and one female patient had only AN and no GU abnormalities, with four having developmental delay. There was similar incomplete phenotypic manifestation among five patients with genotypes commonly described in DDS [9, 32]. Only one patient was defined as incomplete DDS due to her clinical features at diagnosis (age 17.6 months): WT, kidney failure caused by DMS and severe hypertension, without GU anomalies.

Our findings emphasize the importance of considering clinical and pathological findings in addition to the presence of syndromic features in assessing the likelihood that an individual patient with WT carries a *WT1* pathogenic variant. There have been only two major analyses of the prevalence of constitutional *WT1* pathogenic variant in unselected, non-syndromic patients with WT [6, 11]. Among 483 patients with WT enrolled in two large clinical trials in the UK and North America, only 14 (2.9%) had constitutional *WT1* pathogenic variant. Six were aged less than 12 months at diagnosis, eight had GU malformation, four had bilateral tumours, and all with available histology had stromal subtype tumours (3/3). A smaller, single-centre study from the Netherlands reported a frequency of constitutional *WT1* pathogenic variant of 7% (7/97) in non-syndromic WT survivors attending a follow-up clinic [33]. Among these 7 patients, three were under 1 year of age at diagnosis, three had bilateral tumours, 4 of 5 males had GU malformation (cryptorchidism), and 6 of 7 tumours were stromal subtype.

Early identification of underlying *WT1* pathogenic variant in a child with Wilms tumour has potential value in planning the surgical approach to nephron-sparing in relation to the anticipated decline in kidney function. Despite the expected high frequency of kidney failure in patients carrying *WT1* pathogenic variants, to date, only seven (out of 25) patients required chronic dialysis. Of these, three had bilateral nephrectomies performed at an early stage, two for non-responsive bilateral disease and one for unilateral WT and kidney failure due to DMS. One patient had a metachronous relapse 5 years from diagnosis and required complete nephrectomy for tumour control. Of the other three patients, two had bilateral WT and underwent unilateral nephrectomy with contralateral NSS and one patient with unilateral WT received unilateral nephrectomy. The median time between the diagnosis and the start of haemodialysis was 5.56 (0.3–15.9) years.

We investigated whether a difference can be observed between the different *WT1* pathogenic variant subgroups for the rate of deterioration of kidney function at last follow-up. We observed that in the nonsense pathogenic variant subgroup (8 patients), four patients required chronic dialysis (due to CKD in three patients and due to tumour resection in one patient), one developed stage IV CKD (eGFR 21 ml/min/1.73 m^2^), and the other three patients presented albuminuria or were on anti-proteinuric medication. However, this higher rate of kidney function deterioration could also be biased due to the longer follow-up (14 years) reported in this subgroup. Only four patients, three with frameshift pathogenic variants and one with *WT1* deletion, had completely normal kidney parameters at last follow-up. However, the duration of follow-up is short in two patients (1.5 and 3.3 years), longer in the other two (11 and 12 years). All had unilateral WT, three underwent unilateral nephrectomy and one had NSS.

Genotype-phenotype correlation of constitutional *WT1* pathogenic variant has also been studied in children presenting with steroid-resistant nephrotic syndrome (SRNS), with or without Wilms tumour [34, 35]. Long-term preservation of kidney function was found in 25.0% (±3.5%) of 61 children at 10 years from diagnosis of SRNS. Truncating pathogenic variants were associated with a later age at onset of SRNS and splice site pathogenic variants with a slower rate of progression to kidney failure [34]. The constitutional *WT1* pathogenic variant spectrum overlaps with those described here and in other studies of children who present with Wilms tumour rather than nephrotic syndrome. However, the potential for knowledge of the *WT1* pathogenic variant to accurately predict kidney function longevity will require larger numbers of children presenting with cancer or kidney failure to be systematically screened for *WT1* pathogenic variant. With the increasing application of genome sequencing in routine diagnostics, such data are likely to be available in the not too distant future [36].

The strength of this study is that we were able to assemble a large cohort of patients with a rare condition treated at a single centre in whom we have detailed genetic and clinical data. The limitations include the retrospective nature of the data collection, the wide range of length of follow-up for kidney outcomes, and the possibility that covariates (e.g. body mass index) could confound the assessment of kidney function.

In conclusion, our study confirms that *WT1* pathogenic variant is associated with early age of WT diagnosis, GU malformation, bilateral tumours, stromal histology, and ILNR. However, the presence of rhabdomyoblastic differentiation, albuminuria at diagnosis, and persistent hypertension should also raise suspicion of underlying *WT1* pathogenic variant. We would therefore propose that even in the absence of overt clinical features included in the spectrum of *WT1* pathogenic variant, the investigation of a germline *WT1* pathogenic variant should be considered in the presence of bilateral kidney tumours (which may include any combination of WT or precursor lesion), unilateral multifocal disease, age under 12 months, stromal predominance of the lesion in an infant, and any indication of persistent kidney dysfunction or hypertension. Certainly, investigation should be considered if there is presence of any clinical feature within the spectrum of a *WT1* mutation syndrome.

Furthermore, despite the higher frequency of CKD and kidney failure, about two-thirds of the patients in our cohort sustained normal eGFR over a median follow-up period of 9 years. This should guide oncology management regarding the balanced decision about performing NSS without compromising oncological risk. At present, it remains unclear which factors may indicate a higher risk of more rapid deterioration of kidney function. Larger international studies are needed to better categorize these patients according to genotype, phenotype, and the risk of developing kidney failure. Such information would undoubtedly influence decision-making about clinical treatment planning. Since constitutional *WT1* pathogenic variant underlying WT is rare, an international study is required to gather data in a consistent prospective fashion.

## Declarations

### Disclaimer

The views expressed are those of the author(s) and not necessarily those of the NHS, the NIHR, or the Department of Health.

## Abbreviations

AN: aniridia
CKD: chronic kidney disease
DDS: Denys-Drash syndrome
DMS: diffuse mesangial sclerosis
eGFR: estimated glomerular filtration rate
GU: genitourinary
GOSH: Great Ormond Street Hospital
ILNR: intralobar nephrogenic rests
NWTS: National Wilms Tumor Study
NR: nephrogenic rests
NSS: nephron-sparing surgery
PLNR: perilobar nephrogenic rests
SRNS: steroid-resistant nephrotic syndrome
WAGR: WAGR syndrome
WT: Wilms tumour
